# Polycyclic Aromatic Hydrocarbons Pollution Characteristics in Agricultural Soils of the Pearl River Delta Region, China

**DOI:** 10.3390/ijerph192316233

**Published:** 2022-12-04

**Authors:** Haolong Cai, Siyu Yao, Jiahui Huang, Xiongkai Zheng, Jianteng Sun, Xueqin Tao, Guining Lu

**Affiliations:** 1 Provincial Key Laboratory of Petrochemical Pollution Processes and Control, School of Environmental Science and Engineering, Guangdong University of Petrochemical Technology, Maoming 525000, China; 2 College of Resources and Environment, Zhongkai University of Agriculture and Engineering, Guangzhou 510225, China; 3 Department of Environmental Sciences, College of Earth and Environment Sciences, Lanzhou University, Lanzhou 730000, China; 4 Key Laboratory of Ministry of Education on Pollution Control and Ecosystem Restoration in Industry Clusters, Guangdong Provincial Engineering and Technology Research Center for Environmental Risk Prevention and Emergency Disposal, School of Environment and Energy, South China University of Technology, Guangzhou 510006, China

**Keywords:** polycyclic aromatic hydrocarbons, pearl river delta, agriculture soil, spatial distribution, source identification, human health risk

## Abstract

In order to investigate the pollution status of polycyclic aromatic hydrocarbons (PAHs) in the agricultural soil, 240 agricultural soil topsoil samples were collected from nine Pearl River Delta cities from June to September 2019. In addition, 72 samples were collected for vertical soil profiles, which soil profiles were excavated to a depth of 80 cm. After sample preparation, GC-MS was used for the separation of compounds on a HP-5MS quartz capillary column. ArcGIS software was used to map the spatial distribution. Health risk assessment was conducted using USEPA standard. The results showed that the total concentration of 16 PAHs ranged from 43.4 to 5630 ng/g, with an average of 219 ng/g. The spatial distribution showed that most of the seriously polluted areas were in the coastal area, near the port, and there was point source pollution in the Gaoming of Foshan. Vertically distributed display Zhuhai, Jiangmen, Zhaoqing, Shenzhen and Dongguan increased and then decreased from bottom to bottom, showing a low-high-low pattern, the concentration of PAHs in Zhongshan and Foshan decreased with the soil depth, while the concentration of PAHs in Guangzhou and Huizhou was enriched with human activities. The PAHs components in soil samples were mainly medium and high rings (4–6 rings). The analysis of the origin of PAH in soil samples showed that the mixture of incomplete combustion sources of fossil fuels such as coal and biomass and traffic emission sources were the main sources of soil PAHs. A small amount can be attributed to oil sources such as oil spills. The human health risk assessment showed no cancer risk for children, while for adults, may cause a potential risk of cancer, which needs to be noticed. Spearman correlation analysis showed that PAH content was significantly correlated with SOC (*p* < 0.01) and pH (*p* < 0.05). Port transport, road emissions and industrial production make the area a pollution hot topic, and supervision should be strengthened to protect the environment and food safety.

## 1. Introduction

Polycyclic aromatic hydrocarbons (PAHs) are persistent organic pollutants (POPs), which are semi-volatile, and highly toxic [1]. They can migrate over long distances through a variety of environmental media and have carcinogenic, teratogenic, mutagenic, and other toxic effects [2]. PAHs can be adsorbed on tiny particles in the atmosphere and water, and then enter the soil through wet and dry sedimentation or sewage irrigation [3]. PAHs can also enter the soil environment through soil adsorption, plant leaves and root absorption, etc. [4]. The United States Environmental Protection Agency (USEPA) list 16 of these PAHs as priority pollutants and persistent toxic chemical pollutants [5]. PAHs contaminate agricultural all over the world [6]. The density of PAHs in agricultural in a few countries and regions reaches the standard of severe pollution. Due to the wide range of pollution and high hazard nature [7], the pollution of polycyclic aromatic hydrocarbons in the environment has aroused widespread concern.

Due to its hydrophobicity and ester philicity, PAHs are extremely stable and easy to be absorbed by soil and crops and stay there for a long time [8]. According to previous studies, 90% of PAHs in the environment are concentrated in soil. The most common source of pollution in soil comes from atmospheric deposition. Therefore, most PAH from combustion is expected to be confined to the top layer of soil [9].

Not only can PAHs in soil pose risks to humans through accidental ingestion and food contamination, but they may also show toxic activity to different biological elements in the soil environment, such as microorganisms and plants [10]. With the development of industry, the soil pollution in the Pearl River Delta is becoming more and more serious [11]. Increased agricultural and industrial activities may be contributing to rising levels of various chemical pollutants in the environment [12].

Agricultural soil is the most “sensitive” part of the soil environment with respect to human health risk. PAHs in agricultural soils poses a serious risk of introducing these unwanted contaminants into the human food chain [13]. PAHs are primarily harmful to humans through inhalation of PAH-carrying particles, ingestion of contaminated foods from the diet, and direct exposure to contamination [14]. Of great importance for controlling pollution and reducing the risk of human exposure is the identification and distribution of PAHs sources in agricultural soils [15].

According to the research, there have been different degrees of PAHs pollution in the environment soil around the world, such as the Yangtze River Delta region of China [16], Russia [17], Nigeria [18] and other places, although the Pearl River Delta region has also reported related soil PAHs pollution [19], but the investigation area is limited, the sampling points are less, and the interval time is long. Therefore, the basic data of soil PAHs pollution in the PRD area are still insufficient.

This study mainly discusses the following contents: (1) Investigate the level of PAHs pollution in the soil of the PRD and to clarify the composition and pollution characteristics of PAHs in the cities of the PRD. (2) The composition of PAHs was determined by isomer ratio method and the contribution of pollution sources was evaluated. (3) Qualitative assessment of the relationship between SOC, pH, and PAHs pollution. (4) Evaluate the exposure risk of carcinogens in PAHs-contaminated soil for local children and adults. Through the analysis of 240 soil samples in the Pearl River Delta, we can investigate the current situation of PAHs in soils and provide basic data for the pollution of organic pollutants in large regional-scale area and lay a data foundation for the prevention and control of soil pollution in the Pearl River Delta agricultural in the future.

## 2. Materials and Methods

### 2.1. Chemical Reagents

The main reagents used in sample pretreatment are: hexane (chromatography pure), dichloromethane (chromatography pure), 16 kinds of PAHs mixed standard samples: Naphthalene (NAP), acenaphthylene (ANY), dihydroacenaphthylene (ANA), fluorene (FLU), phenanthrene (PHE), anthracene (ANT), fluoranthene (FLT), pyearene (PYEAR), benzo (A) fluoranthene (BaA), Phenanthrene (CHR), benzo (B) fluoranthene (BbFA), benzo (K) fluoranthene (BkFA), benzo (A) pyearene (BaP), NINhydrin Benzene (1, 2, 3—CD) pyearene (IPY), diphenyl and (a, h) anthracene (DBA), benzo[ghi]perylene (BghiP)].

### 2.2. Study Area

The Pearl River Delta includes 9 cities: Dongguan (DG), Jiangmen (JM), Zhongshan (ZS), Huizhou (HZ), Guangzhou (GZ), Shenzhen (SZ), Zhaoqing (ZQ), Zhuhai (ZH) and Foshan. It is located at 112°45′–113°50′ east longitude and 21°31′–23°10′ North latitude, covering an area of about 55,000 km^2^. Located in the South subtropical, subtropical ocean monsoon climate, abundant rainfall, average annual rainfall of 1600–2000 mm, and adequate heat, the main crops for rice, banana, sugarcane, and pineapple.

### 2.3. Sample Collection

Samples were collected from agricultural in 9 cities in the PRD From June to September 2019. Due to the large sampling area, large number of sampling points and insufficient personnel, it took four months to complete the whole sampling from the first point to the last point. In order to ensure that the collected samples were representative, soil from five cores were collected using stainless steel and then composited into one single sample. In addition, 72 samples were collected for vertical soil profiles. The soil profiles were excavated to a depth of 80 cm. Soil samples were collected at 10 cm intervals. A total of 240 soil samples were collected (including 48 soil samples from Guangzhou, 30 soil samples from Shenzhen, 18 soil samples from Dongguan, 30 soil samples from Jiangmen, 16 soil samples from Zhongshan, 28 soil samples from Huizhou, 24 soil samples from Zhaoqing, 19 soil samples from Zhuhai, and 27 soil samples from Foshan). GPS was used to record the longitude and latitude of sampling points.

### 2.4. Sample Preparation

The soil samples were stored at −20 °C until analysis. After drying to remove impurities, such as stone, and then grinding, grinding after 60 mesh sieves.

The extraction method of PAHs of agricultural soil samples was as follows: 6 g soil sample was added to dichloromethane and hexane mixture (1:1, *v*/*v*) for extraction. After ultrasonic and centrifugation (repeated for three times), the supernatant was taken for nitrogen blowing to nearly dry, and the volume was fixed to 3 mL by adding hexane. Finally, after 0.22-micron membrane purification, it was put into sample bottles.

### 2.5. Pollutants Determination and Quality Control

In this study, a meteorological chromatography-mass spectrometer (GCMS-QP2010, Shimadsu, Kyoto, Japan) was used for the separation of compounds on a HP-5MS quartz capillary column (30 m × 0.25 mm inner diameter × 0.25 μm film thickness). The heating procedure is as follows: the initial temperature is 60 °C and maintained for 1 min, then the temperature is raised to 120 °C at the rate of 15 °C/min and maintained for 10 min, and then the temperature is raised to 280 °C at the rate of 10 °C/min and maintained for 15 min. High-purity helium is used as the carrier gas and the flow rate is 1 mL/min. The ion source used in the mass spectrometer was electron bombardment source (EI) and ion monitoring (SIM) mode was used for quantitative analysis of samples. A method-blank sample was included in every batch of 15 samples to control any systematic contamination. In order to ensure the reliability of the experimental data in this study, blank spiked recovery was used to verify the extraction effect, and the recovery rate was higher than 70%.

### 2.6. Health Risk Assessment of PAHs

In order to evaluate the toxicity intensity of PAHs, the concentration of 15 other PAH monomers was converted to BaP equivalent concentration (BaPeq) using benzo (A) pyearene as a representation. According to the cumulative toxic equivalent dose method [20], individual BaPeq of 16 PAH species was calculated by multiplying the measured component concentration with the corresponding toxicity equivalent factor (TEF).

$$\mathrm{CS}={\mathrm{BaP}}_{\mathrm{Eq}}=\sum_{i=1}^{n}C_{i}\times {\mathrm{TEF}}_{i}$$

CS is total BaP_Eq_ concentrations, ∑BaP_Eq_, ng/g;
C*_i_* is the concentration of the ith kind of PAH, ng/g;

In this study, the USEPA Standard model Estimated proliferative Lifetime Cancer Risk (ILCR) 12 was used to assess the potential health risks of exposure to PAHs in the soil in adults and children in the Pearl River Delta region. Typically, humans are exposed to soil contaminants through three routes: ingestion, inhalation, and skin absorption, and the total risk is the sum of the individual risks associated with each route of exposure [10]. The ILCR of the three pathways was calculated as follows:

$${\mathrm{ILCR}}_{\mathrm{ingestion}}=\frac{\mathrm{CS}\times {\mathrm{CSF}}_{\mathrm{ingestion}}\times \sqrt[{3}]{\frac{\mathrm{BW}}{70}}\times {\mathrm{IR}}_{\mathrm{soil}}\times \mathrm{EF}\times \mathrm{ED}}{\mathrm{BW}\times \mathrm{AT}\times 10^{6}}$$

$${\mathrm{ILCR}}_{\mathrm{dermal}}=\frac{\mathrm{CS}\times {\mathrm{CSF}}_{\mathrm{dermal}}\times \sqrt[{3}]{\frac{\mathrm{BW}}{70}}\times \mathrm{SA}\times \mathrm{AF}\times \mathrm{ABS}\times \mathrm{EF}\times \mathrm{ED}}{\mathrm{BW}\times \mathrm{AT}\times 10^{6}}$$

$${\mathrm{ILCR}}_{\mathrm{inhalation}}=\frac{\mathrm{CS}\times {\mathrm{CSF}}_{\mathrm{inhalation}}\times \sqrt[{3}]{\frac{\mathrm{BW}}{70}}\times {\mathrm{IR}}_{\mathrm{air}}\times \mathrm{EF}\times \mathrm{ED}}{\mathrm{BW}\times \mathrm{AT}\times \mathrm{PEF}}$$

CSF is Oncogenic slope factor, CSF_ingestion_ value 7.3, CSF_dermal_ value25, CSF_inhalation_ value 3.85, (mg·kg^−1^·d^−1^)^−1^;
IR_soil_ is Soil absorption rate, adult value 100 children value 200, mg·d^−1^;
IR_air_ is Air absorption rate, adult value 200 children value 5, m^3^·d^−1^;
EF is exposure frequency, value 350, day·year^−1^;
ED is exposure duration, adult value 24 children value 6, year;
BW is Body weight, adult value 64 children value 15, kg;
SA is soil surface area, adult value 5000 children value 1800, cm^2^·d^−1^;
AF is skin adhesion factor, value 1, mg·cm^2^;
ABS is skin adsorption rate, value 0.1;
AT is skin adsorption rate, value 70, year;
PEF is particle emission factor, value 1.32 × 10^9^, m^3^/kg;

### 2.7. Correlation Analysis

This study measured soil organic carbon (SOC) and pH values for correlation analysis. The test method of SOC is referred to as the potassium dichromate volumetric method, or dilution thermal method in Soil Agrochemical Analysis. The specific methods are as follows:

Next, accurately add 1 mol·L^−1^ (1/6K_2_Cr_2_O_7_) 10 mL solution to the soil sample, rotate the bottle to mix evenly, and then add 20 mL H_2_SO_4_. Slowly rotate the triangle bottle for 1 min. The reagent was mixed to ensure the full interaction between the reagent and the soil, and was placed on the asbestos plate for about 30 min, diluted with 250 mL water, and added with 3–4 drops of shoreline indicator. The color of the solution changed from green to dark green near the end point of titration with 0.5 mol·L^−1^ FeSO_4_ standard solution, and gradually added FeSO_4_ until brick red was generated.

The calculation formula is as followed:

$$\mathrm{SOC}\left(g\cdot {\mathrm{kg}}^{-1}\right)=\frac{c\left(V_{0}-V\right)\times 10^{-3}\times 3.0\times 1.33}{\mathrm{Drying}\;\mathrm{soil}\;\mathrm{weight}}\times 1000$$

Blank assays (i.e., without soil samples) were made in the same way.
1.33 is oxidation correction factor.
c is Concentration of 0.5 mol/L FeSO_4_ standard solutions.

### 2.8. Data Analysis

Origin 8.5, Microsoft Excel 2016 and SPSS 22.0 were used for statistical analysis and chart making of the measured data. Spearman correlation analysis was used to evaluate the relationship between soil pollutant concentration, soil organic carbon (SOC) and pH. Bigemap and ArcGIS 10.5 were used to map the spatial distribution of 16 PAHs in the soil of the PRD.

## 3. Results and Discussion

### 3.1. Levels of PAHs in Soil

The statistical results of the concentration range of PAHs in the agricultural soil of the PRD were shown in Table 1. Total concentrations of ∑16PAHs in topsoil ranged from 43.4 to 5630 ng/g, with an average of 219 ng/g, which means the distinct difference. The average concentration of BbFA (30.5 ng/g) was higher than the others among all PAHs. According to the average concentration, the most prevalent PAHs were ranked as: 5 rings > 4 rings > 6 rings > 3 rings > 2 rings.

Furthermore, except NAP, the coefficient of variation of the other 15 PAHs was lower than 100%, and the coefficient of variation was 40.62%, indicating that the dispersion of PAHs in the PRD was low, and the content of PAHs in different regions had little difference. The detection rates of 16 PAHs were all above 93%, indicating that PAHs were widely present in agricultural soil, this may be due to historical residual reasons.

Soil pollution was evaluated by Maliszewska-Kordybach’s proposed grading standard [21]. This evaluation method divides the PAHs pollution in soil into four levels: no pollution (∑PAHs < 200 ng/g), mild pollution (200 ng/g < ∑PAHs < 600 v), moderate pollution (600 ng/g < ∑PAHs < 1000 ng/g), and severe pollution (1000 ng/g < ∑PAHs). In the PRD region, the number of unpolluted, mildly polluted, moderately polluted and seriously polluted soil samples was 187, 40, 7 and 5, respectively. They accounted for 78.24%, 16.74%, 2.93% and 2.09% of the total analyzed samples, respectively, and 21.76% of the soil samples were contaminated by different degrees of PAHs, which should be paid attention to by the government. Among the three sampling points with the most serious PAHs pollution, one was in the west of the region, and the other two were located at the Pearl River port, with concentrations of 5630 ng/g, 4940 ng/g and 3300 ng/g, respectively. Compared with previous studies on PAHs content in agricultural soil in the PRD [19], accounted for 66.9%, 25%, 6.2% and 1.9% of the total analyzed samples respectively, and 33.4% of the soil samples were contaminated by different degrees of PAHs, the proportion of contaminated agricultural soil in the Pearl River Delta has decreased, which indicates that the past decade has played a positive role in soil pollution control, but local pollution is more severe than before, which may be related to emissions from factories around the sampling sites.

Compared to the previous studies in other cities of China, the concentration of PAHs in the PRD region is lower than that of other regions in China, which indicates that the emission of PAHs has been limited in the past ten years. Meanwhile, due to the regional diversity, the difference of PAHs concentration in different urban soils was affected by the temperature, climate, soil organic matter and environment in different regions (Table 2). Compared to previous studies conducted in other countries, the concentration of PAHs in the PRD was only slightly higher than that in Switzerland, but the concentration in the Pearl River Delta was more concentrated over a smaller span (Table 2). Seasonal changes will have an impact on pollution. It was found that the concentration of PAHs in karst mining area soil presented a seasonal pattern of that in winter > spring > autumn > summer [22]. In Yinma River Basin, Chen et al. found that the concentration of 16 PAHs in November is a little bit higher than that in May and August, and the concentration of 16 PAHs in August is the lowest [23]. The reason for the difference may be due to the rain during summer resulted in a lesser accumulation of pollutants. Moreover, degradation of PAHs becomes enhanced under high temperature and strong sunlight during summer. In the PRD, the erosion of soil by rain will accelerate the migration and transformation of PAHs in soil [24], resulting in a high degree of soil weathering and leaching and increased soil microbial activity, thus accelerating the degradation of PAHs. However, in this study we did not investigate seasons as an influencing factor, which will be investigated in the future.

### 3.2. Spatial Distribution of PAHs in Soil

**Figure 1:**
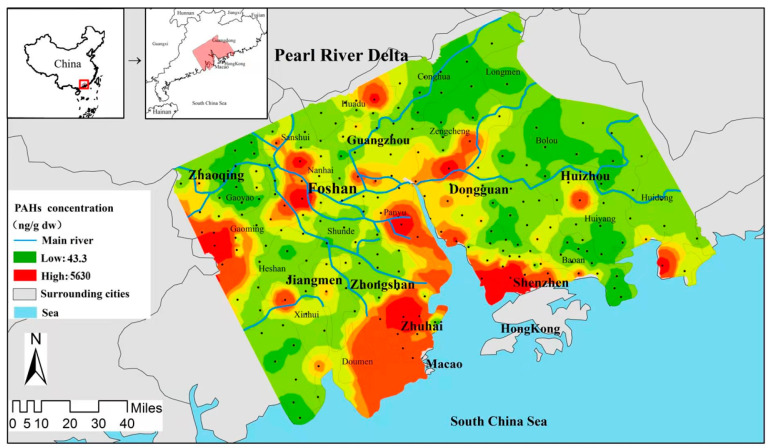
The spatial distribution map of PAHs in the soil from the PRD, China.

The spatial distribution map of PAHs was shown in the following Figure 1. The distribution of PAHs in the PRD has obvious regional characteristics, and high concentration sampling sites are mainly located in the western part of the PRD region and Pearl River ports, such as the western part of Foshan, the western part of Jiangmen, the southern part of Guangzhou, the southern part of Shenzhen, and Zhuhai. There may be point source pollution in the Gaoming area of western Foshan, according to previous research reports, PAHs pollution in agricultural soils near ports in the PRD region is in different degrees, which is consistent with the results obtained in this study, which may be caused by the ship transportation process.

In contrast, except for central Foshan, southern Huizhou and a small part of northern Guangzhou, the concentration of PAHs in most areas of the PRD is low, and only a few sites are seriously polluted (>1000 ng/g). The sampling point with higher concentrations of PAHs were mostly located next to roads (highways, expressways) and near factories.

According to the distribution of topsoil PAHs content by city, the soil PAHs pollution of the PRD ports (Zhuhai, Shenzhen and southern Guangzhou) was significantly higher than that of Huizhou in the northeast of the PRD, Jiangmen and Zhaoqing in the west, and the northern part of Guangzhou. The Pearl River port cities in the PRD are the most economically developed areas. The impact of human activities on the natural environment is reflected by regional differences in soil PAHs pollution.

In the Pearl River Delta region, the temperature is usually warm, the air humidity is high, and the ultraviolet radiation is strong, which will affect the degradation of PAHs in soil [40]. Therefore, the content of PAHs in most agricultural soils in the Pearl River Delta is not high, and the concentration is high [41].

The distribution of PAHs in soil also indicates that PAHs in soil in the Pearl River Delta region is strongly influenced by human activities. The PAHs emission increases in port area due to the heavy exhaust of large motor vehicles and the dense population [42].

### 3.3. Vertical Distribution of PAHs in Soil Profiles

**Figure 2:**
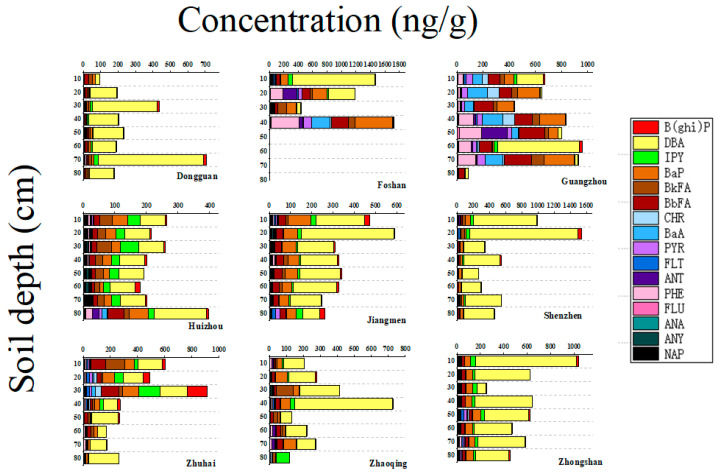
The vertical distribution of PAHs in agricultural soils in the PRD, China.

The vertical concentration and distribution of PAHs in soils at different depths in the PRD were mapped and the results are shown in Figure 2. PAHs residues are different in different cities, which may be relevant to local agricultural practices and industry. The content of PAHs in Zhuhai, Jiangmen, Zhaoqing, Shenzhen and Dongguan increased and then decreased from bottom to bottom, showing a low-high-low pattern, which indicated that the content of PAHs in soil increased from a relatively low value with the strengthening of human activities, and then decreased with the enhancement of social environmental awareness and improvement of energy structure. The concentration of PAHs in Zhongshan and Foshan decreased with the soil depth, while the concentration of PAHs in Guangzhou and Huizhou was enriched. This is because high molecular weight PAHs mainly exist in the form of particles, which are not easy to migrate in the soil and will always remain in the soil [43].

### 3.4. Composition Analysis of PAHs in Soil

The average proportion of PAHs in the PRD topsoil was 15.25% in the low ring, 31.38% in the central ring, and 53.36% in the high ring. PAHs in the soil of the PRD are mainly in the middle-high ring. That is to say, the pollution of agricultural soil in the PRD is dominated by high molecular weight PAHs, which is mainly because PAHs are a kind of semi-volatile organic compounds [44]. With the increase of molecular weight, their volatility gradually decreases, and their existing form gradually changes from gaseous to granular state. Low molecular weight PAHs are easy to be volatilized and photolysis to atmospheric environment, while high molecular weight PAHs with higher *K*_ow_ tend to be distributed on soil particles [24]. It also explains why PAHs are enriched at the bottom of the soil.

The percentage of medium and high ring PAHs in total PAHs is 84.74%. The main source of PAHs in agricultural soils in the Pearl River Delta region is fossil fuels (insufficiently burned). Due to the low saturated vapor pressure, high cyclic PAH is particle reactive and does not migrate easily [41]. Compared with low cyclic PAHs, high cyclic PAHs degrade slowly and have higher toxicity and longer lasting. Therefore, environmental control and pollution mitigation of PAHs in agricultural soil in the Pearl River Delta region should be carried out.

As for the ratio relationship between the number of PAHs generated by pollution sources, some studies believe that high temperature combustion sources will produce mainly high ring PAHs pollution, and their light to heavy ratio (LMW/HMW) is less than 1, while oil sources will produce mainly low ring PAHs pollution, (LMW/HMW) is more than 1. It can be preliminarily determined that the pollution mainly comes from the incomplete combustion of fossil fuels such as coal, biomass, and gasoline.

### 3.5. Preliminary Source Analysis of PAHs in Soil

In addition to natural causes, PAHs mainly come from human activities and energy utilization processes. The incomplete combustion products of coal and petroleum are the main sources of PAHs. PAHs from the above sources enter the soil mainly through the following two ways: first, PAHs settle on the soil surface along with atmospheric particles. Second, the PAHs in the atmosphere enter the soil with rain in the form of gas. Fluoranthene (FLT) and pyearene (PYEAR) can be produced by incomplete combustion of mineral oil, coal, and biomass, and coal tar itself contains both compounds [45]. However, the ratio of fluoranthene to pyearene in PAHs from incomplete combustion and mineral oil is different.

Based on the thermodynamic stability of PAHs isomers, the diagnostic ratio of PAHs is widely used for source estimation [46], and the main diagnostic ratios of PAHs contained FLA/(FLA + PYEAR), and IPY/(IPY + BghiP). In this study, FLA/(FLA PYEAR) and IPY/(IPY + BghiP) were selected to identify the local emission sources of soil PAHs. The FLT/(FLT + PYEAR) values at the range of <0.4, 0.4–0.5, and >0.5 indicate that the main pollution source were oil source, fossil fuel combustion, and biomass combustion. Furthermore, the IPY/(IPY + Bghip) values at the range of <0.2, 0.2–0.35, and >0.35 suggest the oil source pollution, combustion of petroleum products, and biomass combustion [2].

**Figure 3:**
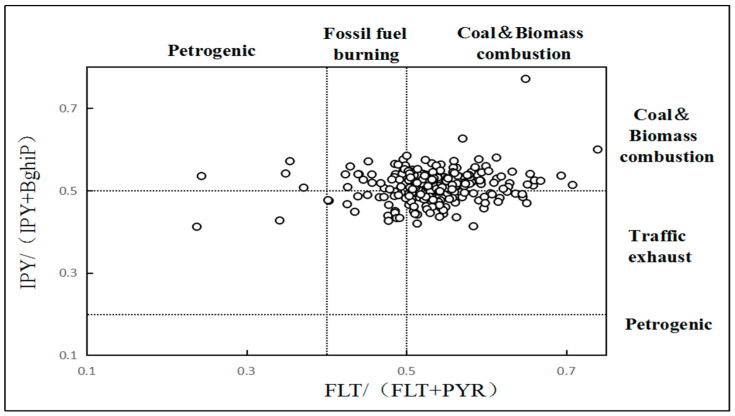
PAH diagnostic ratios in the studied soil samples.

The FLT/(FLT + PYEAR) values ranges from 0.23 to 0.74, with an average of 0.53. The ratio of IPY/(IPY + Bghip) ranged from 0.41 to 0.77, with an average of 0.51 (Figure 3). The results of diagnostic ratios show that the major contribution of PAHs was the mixture of incomplete combustion sources of fossil fuels such as coal and biomass and transportation emission sources. Apart from that, oil leakage was also attributed to PAHs pollution. The results show that origins of PAHs contamination were complicated, which was the result of interleaving and accumulation of multiple pollution pathways.

### 3.6. Health Risk Assessment

**Figure 4:**
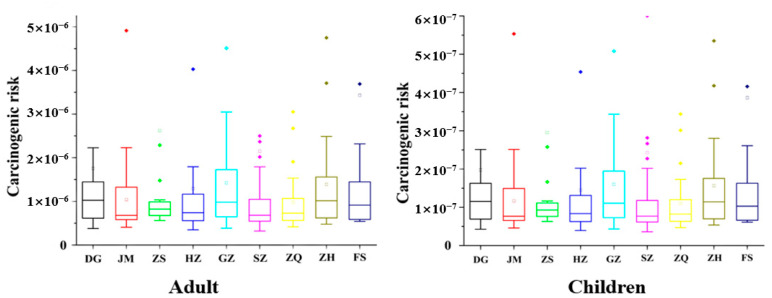
The carcinogenic risk assessment of PAHs for local adults and children.

On the basis of EPA recommended guidelines, ILCR values < 10^−6^, 10^−6^ to 10^−4,^ and >10^−4^ indicate negligible risk, potential risk, and high risk of cancer [21], respectively. The ILCR values estimated from the average concentration of 16 priority PAHs in the surface soil of the Pearl River Delta region are shown in the Figure 4.

The results showed that the 90th percentile cumulative probability of ILCR for local adults and children exposed to average concentrations of PAHs in surface soil was 2.36 × 10^−6^ and 2.66 × 10^−7^, respectively. The mean ILCR data fell in the range from 10^−6^ to 10^−5^, which meant that exposure of local adults and children to PAHs in topsoil may cause a potential risk of cancer, especially for local adults, possibly due to the fact that a longer duration of contaminant in adults than in children.

The cancer risk for local adults and children through the three routes of exposure appears in the following order: skin contact > ingestion > inhalation. In addition, the 90% cumulative probability of cancer risk by inhaling PAHs in topsoil particles is negligible, it is significantly lower than the other two pathways.

In conclusion, the accumulation of PAHs in the Pearl River Delta region has reached a level that may harm human health, and the concentration of PAHs in industrially developed areas may be higher. Therefore, it is urgent for us to take measures, so as to provide data support for the protection of soil safety and people’s safety.

### 3.7. Correlations between Soil Properties and PAHs

The transfer of PAHs in soil was realized through diffusion and water seepage [30]. Therefore, the distribution characteristics and migration rules of PAHs in soil may be affected not only by the nature of pollutants themselves, but also by soil physical and chemical properties such as pH, temperature and organic matter content. The correlation between soil physical and chemical properties and distribution characteristics of PAHs in each region of the PRD will be analyzed and determined in this study.

Previous studies have suggested that there should be a lack of correlation [47], at least until equilibrium is reached, in environments where new PAH pollution is introduced. Spearman correlation analysis showed that PAH content in PRD soil was significantly positive correlated with SOC (*p* < 0.01) when SOC content was 0.08–46.36 ng/kg. The geographic distribution pattern of PAHs is close to steady state and is in equilibrium with soil properties, which is shown by a good correlation between PAHs and organic carbon concentration.

In this study, soil pH ranged from 4.50 to 8.87. Spearman correlation analysis showed that PAH content in PRD soil was significantly positive correlated with pH (*p* < 0.05). The change of PAHs concentration in soil may be because of pH on the composition and structure of humic acid in soil.

### 3.8. Ecological Risk Assessment

Organic pollutants have low effect interval (ERL) and middle effect interval (ERM) of ecological risk. At present, ERL/ERM has been widely used in soil and sediment. According to the data of pollutant content, the effect interval of pollutant content is judged. If the concentration was lower than the low value of effect interval (ERL), the probability of negative ecological effect was less than 10%. If the concentration was in the middle between the low value of the effect interval and the middle value of the effect interval (ERM), it was considered that there were occasional negative ecological effects. If the concentration was higher than the median effect interval (ERM), it was considered that the probability of a negative ecological effect was greater than 75%.

According to the effect interval low-median method, the effect interval of 240 soil samples in the PRD was divided into different PAHs. Among the 240 soil samples, the concentrations of naphthalene, fluoranthene, and pyearene were lower than the low value of the effect interval. The concentration of the other 13 kinds of PAHs were all in the region between the low value and the median value of the effect interval, so it was considered that the probability of a negative ecological effect was less than 10% or a negative ecological effect occurred occasionally.

Through the low/median effect interval method, the progress of pollution in the PRD can be roughly described, from less than 10% probability of causing negative environmental effects to occasionally causing negative environmental effects. From no pollution to light pollution, some have changed to heavy pollution. A series of measures should be put forward to reduce PAHs pollution and prevent pollution from going to extremes.

## 4. Limitation

Although this study provided the concentration data of PAHs in soils and provide basic data for the pollution of organic pollutants in the Pearl River Delta, there are still limitations. The main limitations are as follows: (1) Seasonal difference has influence on pollution. In this study, the influence factor of season has not been studied. (2) The sampling did not according to difference land use types, no distinction between farmland, forest land, grassland. (3) The analysis of physical and chemical properties of soil is not comprehensive enough. Only pH and SOC were analyzed, but other properties such as total nitrogen, total phosphorus and total potassium were not analyzed.

## 5. Conclusions

(1) According to agricultural sampling, the number of unpolluted, mildly polluted, moderately polluted, and seriously polluted soil samples in the topsoil of the PRD region were 187, 40, 7, and 5, respectively. 21.76% of the soil samples were contaminated by different degrees of PAHs. The total concentration of 16 PAHs ranged from 43.4 to 5630 ng/g, with an average of 219 ng/g. Soil PAHs in the Pearl River Delta was a mainly medium and high rings, accounting for an average of 84.7%.
(2) PAH in agricultural soil samples in the PRD region mainly comes from the mixture of incomplete combustion sources of fossil fuels such as coal and biomass and transportation emission sources. PAHs input from petroleum is a relatively small part. PAH content in topsoil was significantly positive correlated with SOC (*p* < 0.01) and pH (*p* < 0.05).
(3) In this study, the ILCR values of PAHs were estimated to be 10-6 to 10-5, indicating that exposure to surface soil PAHs in local adults and children in the Pearl River Delta region may bring potential cancer risks. In addition, the exposure risk stems mainly from PAH ingestion in topsoil and skin contact.

Therefore, in the context of agricultural safety and ecosystem health, future research should focus on reducing the accumulation of PAHs in soil. On this basis, we suggest that remediation technology, such as phytoremediation and microbial joint remediation, be used to restore contaminated soils and the studied area should also be continuously monitored.

## Figures and Tables

**Table 1:** The concentration (ng/g, dw) of 16 kinds of PAHs in topsoil of the PRD (*n* = 240).

Compound	Benzene Rings	Min ng/g	Max ng/g	Mean ng/g	CV /%	Detection Rate/%
NAP	2	0.2	51.6	7.08	103.4	100
ANY	3	ND	63.4	1.36	25.3	99.6
ANA	3	0.1	19.4	1.10	56.3	100
FLU	3	ND	69.3	4.60	64.8	99.6
PHE	3	ND	446	13.1	32.8	99.6
ANT	3	ND	390	6.13	21.9	99.6
FLT	4	ND	599	15.7	28.1	99.6
PYEAR	4	ND	471	13.7	31.1	99.6
BaA	4	5.9	580	20.3	40.5	100
CHR	4	ND	531	19.2	40.2	99.6
BbFA	5	5.19	843	30.5	40.3	100
BKFA	5	5.93	287	15.9	51.8	100
BaP	5	5.23	664	25.1	39.9	100
DBA	5	ND	200	6.88	35.4	99.2
IPY	6	0.91	664	20.2	30.8	93.8
B(ghi)p	6	ND	497	18.1	31.9	98.8
∑16PAHs		43.4	5630	219	40.6	100

ND: not detected. CV: Coefficient of Variation.

**Table 2:** The soil PAHs concentrations in soils from different areas of the world.

Regions	Year of Sampling	Range ng/g	Mean ng/g	References
Pearl River Delta, China	2005	58~3077	315	[19]
Shanghai, China	2006	347~17,900	3290	[25]
Beijing, Tianjin, China	2007	31.6~1480	336	[26]
Taizhou City, China	2016	77~6631	1362	[27]
Yangtze River Delta	2016	10.1~3060	267	[16]
Changchun, China	2019	46.6~8871	1480	[28]
Poyang Lake in China	2022	45.1~3158	531.4	[29]
Shanxi Province, China	2022	22.1~1338	224	[30]
Lakes in Eastern China	2022	122.9~743.4	275	[31]
Japan	1959–2002	52.9~2180	496	[32]
Switzerland	1995–1999	32~8460	163	[6]
Britain	2006	40.4~14,100	946	[33]
South Korea	2010	65~12,000	960	[34]
Iran	2015	75.8~15,508	1733	[35]
German	2015	105~14,900	1448	[36]
USA	2016	43~30,428	3227	[37]
Southern Russia	2017	147~4787	1730	[17]
Montenegro	2019	60.8~1457	271	[38]
India	2020	2223~11,266	5867	[39]
Pearl River Delta	2019	43.4~5630	219	This study

## References

[1] Liu G.Q., Yu L.L., Li J., Liu X., Zhang G. (2011). PAHs in soils and estimated air-soil exchange in the Pearl River Delta, South China. Environ. Monit. Assess..

[2] Li H., Lai Z., Zeng Y., Gao Y., Yang W., Mai Y., Wang C. (2021). Occurrence, source identification, and ecological risk assessment of polycyclic aromatic hydrocarbons in sediments of the Pearl River Delta, China. Mar. Pollut. Bull..

[3] Li A., Beek T.A., Schubert M., Yu Z., Schiedek T., Schuth C. (2019). Sedimentary archive of Polycyclic Aromatic Hydrocarbons and perylene sources in the northern part of Taihu Lake, China. Environ. Pollut..

[4] Tsibart A.S., Gennadiev A.N. (2013). Polycyclic aromatic hydrocarbons in soils: Sources, behavior, and indication significance (a review). Eurasian Soil Sci..

[5] Song Y.T., Hao R., Peng S.L., Wan H.F. (2013). Polycyclic Aromatic Hydrocarbons in different soils and vegetables from the Pearl River Delta, South China. Environ. Eng. Manag. J..

[6] Desaules A., Ammann S., Blum F., Brandli R.C., Bucheli T.D., Keller A. (2008). PAH and PCB in soils of Switzerland—Status and critical review. J. Environ. Monitor..

[7] Xiao Y., Tong F., Kuang Y., Chen B. (2014). Distribution and source apportionment of polycyclic aromatic hydrocarbons (PAHs) in forest soils from urban to rural areas in the Pearl River Delta of Southern China. Int. J. Environ. Res. Public Health.

[8] Liu B.L., Li M., Hua X.Y., Dong D.M., Dong W.H. (2013). Distribution and Source Identification of Polycyclic Aromatic Hydrocarbons Residues in Different Types of Topsoil Collected from Jilin Province, China. Asian J. Chem..

[9] Tobiszewski M., Namiesnik J. (2012). PAH diagnostic ratios for the identification of pollution emission sources. Environ. Pollut..

[10] Cai C., Li J., Wu D., Wang X., Tsang D.C., Li X., Sun J., Zhu L., Shen H., Tao S. (2017). Spatial distribution, emission source and health risk of parent PAHs and derivatives in surface soils from the Yangtze River Delta, eastern China. Chemosphere.

[11] Ma Y., McGree J., Liu A., Deilami K., Egodawatta P., Goonetilleke A. (2017). Catchment scale assessment of risk posed by traffic generated heavy metals and polycyclic aromatic hydrocarbons. Ecotoxicol. Environ. Saf..

[12] Zhu Y., Tao S., Sun J., Wang X., Li X., Tsang D.C.W., Zhu L., Shen G., Huang H., Cai C. (2019). Multimedia modeling of the PAH concentration and distribution in the Yangtze River Delta and human health risk assessment. Sci. Total Environ..

[13] Gao M., Dong Y., Zhang Z., Song Z. (2020). Effect of dibutyl phthalate on microbial function diversity and enzyme activity in wheat rhizosphere and non-rhizosphere soils. Environ. Pollut..

[14] Zhang Y., Wei L.F., Li Y., Du S.X., Liu S.Y., Liu X.Y. (2016). Distribution and Health Risk Assessment of Polycyclic Aromatic Hydrocarbons in Surface Water of the Xijiang River, China. Pearl River.

[15] Liu Y.Y., PAN C., Li Q.W., Xu X., Su Y.X. (2021). Characteristics and health risk assessment of PAHs in particulate matter of mainroads in Kaifeng in summer and autumn. Environ. Chem..

[16] Sun J.T., Pan L.L., Zhan Y., Lu H.N., Tsang D.C.W., Liu W.X., Wang X.L., Li X.D., Zhu L.Z. (2016). Contamination of phthalate esters, organochlorine pesticides and polybrominated diphenyl ethers in agricultural soils from the Yangtze River Delta of China. Sci. Total Environ..

[17] Sazykin I.S., Minkina T.M., Khmelevtsova L.E., Antonenko E.M., Azhogina T.N., Dudnikova T.S., Sushkova S.N., Klimova M.V., Karchava S.K., Seliverstova E.Y. (2021). Polycyclic aromatic hydrocarbons, antibiotic resistance genes, toxicity in the exposed to anthropogenic pressure soils of the Southern Russia. Environ. Res..

[18] Okoye E.A., Bocca B., Ruggieri F., Ezejiofor A.N., Nwaogazie I.L., Domingo J.L., Rovira J., Frazzoli C., Orisakwe O.E. (2021). Concentrations of polycyclic aromatic hydrocarbons in samples of soil, feed and food collected in the Niger Delta region, Nigeria: A probabilistic human health risk assessment. Environ. Res..

[19] Yang G.Y., Zhang T.B., Guo Z.X., Wan H.F., Luo W., Gao Y.X. (2007). The Source and Distribution Characteristics of Polycycclic Aromatic Hydrocarbons in Agricultural Soils in the Pearl River Delta. Environ. Chem..

[20] Yang W., Lang Y., Li G. (2014). Cancer risk of polycyclic aromatic hydrocarbons (PAHs) in the soils from Jiaozhou Bay wetland. Chemosphere.

[21] Lv J., Shi R., Cai Y., Liu Y. (2010). Assessment of polycyclic aromatic hydrocarbons (PAHs) pollution in soil of suburban areas in Tianjin, China. Bull Environ. Contam. Toxicol..

[22] An X.J., Li W., Lan J.C., Di X.Y., Adnan M. (2022). Seasonal co-pollution characteristics of parent-PAHs and alkylated-PAHs in karst mining area soil of Guizhou, Southwest China. Front. Environ. Sci..

[23] Chen Y.N., Zhang J.Q., Zhang F., Liu X.P., Zhou M. (2018). Contamination and health risk assessment of PAHs in farmland soils of the Yinma River Basin, China. Ecotoxicol. Environ. Saf..

[24] Yao S.Y., Huang J.H., Zhou H.J., Cao C.T., Ai T., Xing H.H., Sun J.T. (2022). Levels, Distribution and Health Risk Assessment of Organochlorine Pesticides in Agricultural Soils from the Pearl River Delta of China. Int. J. Environ. Res. Public Health.

[25] Jiang Y.F., Wang X.T., Wang F., Jia Y., Wu M.H., Sheng G.Y., Fu J.M. (2009). Levels, composition profiles and sources of polycyclic aromatic hydrocarbons in urban soil of Shanghai, China. Chemosphere.

[26] Wang W., Massey Simonich S.L., Xue M., Zhao J., Zhang N., Wang R., Cao J., Tao S. (2010). Concentrations, sources and spatial distribution of polycyclic aromatic hydrocarbons in soils from Beijing, Tianjin and surrounding areas, North China. Environ. Pollut..

[27] Su M., Zhu Z., Li T., Jin J., Hu J. (2022). Levels, profiles and potential human health risks of brominated and parent polycyclic aromatic hydrocarbons in soils around three different types of industrial areas in China. Sci. Total Environ..

[28] Zhao W., Li J., Lu J.L., Lan T., Zang L.B., Guo J.K., Li T. (2022). Polycyclic Aromatic Hydrocarbons (PAHs) in Main urban area Soils of Changchun, Northeast China: Status, Sources, and Potential Toxic Risk Assessment. Pol. J. Environ. Stud..

[29] Chen C.L., Zeng H.Q., Gong X.F., Li J., Wang L.Q. (2022). PAHs Source Identification in Sediments and Surrounding Soils of Poyang Lake in China Using Non-Negative Matrix Factorization Analysis. Land.

[30] Ji L., Li W.W., Li Y., He Q.S., Bi Y.H., Zhang M.H., Zhang G.X., Wang X.M. (2022). Spatial Distribution, Potential Sources, and Health Risk of Polycyclic Aromatic Hydrocarbons (PAHs) in the Surface Soils under Different Land-Use Covers of Shanxi Province, North China. Int. J. Environ. Res. Public Health.

[31] Zhao Z.W., He W., Wu R.L., Xu F.L. (2022). Distribution and Relationships of Polycyclic Aromatic Hydrocarbons (PAHs) in Soils and Plants near Major Lakes in Eastern China. Toxics.

[32] Honda K., Mizukami M., Ueda Y., Hamada N., Seike N. (2007). Residue level of polycyclic aromatic hydrocarbons in Japanese paddy soils from 1959 to 2002. Chemosphere.

[33] Heywood E., Wright J., Wienburg C.L., Black H.I.J., Long S.M., Osborn D., Spurgeon D.J. (2006). Factors influencing the national distribution of polycyclic aromatic hydrocarbons and polychlorinated biphenyls in British soils. Environ. Sci. Technol..

[34] Kwon H.O., Choi S.D. (2014). Polycyclic aromatic hydrocarbons (PAHs) in soils from a multi-industrial city, South Korea. Sci. Total Environ..

[35] Mohit A., Keshavarzi B., Moore F. (2019). Polycyclic aromatic hydrocarbons (PAHs) in urban soils of Ahvaz metropolis; contamination, composition, distribution, potential sources, and cancer risk. Hum. Ecol. Risk Assess..

[36] Aichner B., Bussian B.M., Lehnik-Habrink P., Hein S. (2015). Regionalized concentrations and fingerprints of polycyclic aromatic hy-drocarbons (PAHs) in German forest soils. Environ. Pollut..

[37] Liu Y.G., Gao P., Su J., da Silva E.B., de Oliveira L.M., Townsend T., Xiang P., Ma L.N.Q. (2019). PAHs in urban soils of two Florida cities: Background concentrations, distribution, and sources. Chemosphere.

[38] Bigović M., Đurović D., Nikolić I., Ivanović L., Bajić B. (2022). Profile, Sources, Ecological and Health Risk Assessment of PAHs in Agricultural Soil in a Pljevlja Municipality. Int. J. Environ. Res..

[39] Ambade B., Sethi S.S., Chintalacheruvu M.R. (2022). Distribution, risk assessment, and source apportionment of polycyclic aromatic hydrocarbons (PAHs) using positive matrix factorization (PMF) in urban soils of East India. Environ. Geochem. Health.

[40] Ma Y., Cheng L., Ruan Z.Y., Shi P.F., Lu C.J., Yun X.T., Li L.Y., Xu Y.Q., Shi Y. (2021). Polycyclic Aromatic Hydrocarbons in Surface Soil of China (2000–2020): Temporal and Spatial Distribution, Influencing Factors. Environ. Chem..

[41] Zhang P., Chen Y.G. (2017). Polycyclic aromatic hydrocarbons contamination in surface soil of China: A review. Sci. Total Environ..

[42] Suman S., Sinha A., Tarafdar A. (2016). Polycyclic aromatic hydrocarbons (PAHs) concentration levels, pattern, source identification and soil toxicity assessment in urban traffic soil of Dhanbad, India. Sci. Total Environ..

[43] Krzebietke S.J., Mackiewicz-Walec E., Sienkiewicz S., Wierzbowska J., Zaluski D., Borowik A. (2022). Polycyclic Aromatic Hydrocarbons in Soil at Different Depths under a Long-Term Experiment Depending on Fertilization. Int. J. Environ. Res. Public Health.

[44] Wang J.Z., Zhu C.Z., Chen T.H. (2013). PAHs in the Chinese environment: Levels, inventory mass, source and toxic potency assessment. Environ. Sci.-Proc. Impacts.

[45] Wang H., Yang Y.T., Walker T.R., Wang Y.G., Wu H., Wang X.X., Luo Q. (2022). Characterization, source apportionment, and risk assessment of polycyclic aromatic hydrocarbons (PAHs) in urban soils from 23 cities in China. Environ. Sci. Pollut. Res. Int..

[46] Chung M.K., Hu R., Cheung K.C., Wong M.H. (2007). Pollutants in Hong Kong soils: Polycyclic aromatic hydrocarbons. Chemosphere.

[47] Agarwal T., Khillare P.S., Shridhar V., Ray S. (2009). Pattern, sources and toxic potential of PAHs in the agricultural soils of Delhi, India. J. Hazard. Mater..

